# Impact of the gut microecology on *Campylobacter* presence revealed by comparisons of the gut microbiota from chickens raised on litter or in individual cages

**DOI:** 10.1186/s12866-021-02353-5

**Published:** 2021-10-22

**Authors:** Wei Yan, Qianqian Zhou, Zhongyang Yuan, Liang Fu, Chaoliang Wen, Ning Yang, Congjiao Sun

**Affiliations:** 1 Poultry Science Laboratory, College of Animal Science and Technology, China Agricultural University, Beijing, 100193 China; 2 National Engineering Laboratory for Animal Breeding and Key Laboratory of Animal Genetics, Breeding and Reproduction, Ministry of Agriculture and Rural Affairs, Beijing, 100193 China

**Keywords:** *Campylobacter*, Chicken, Gut microbiome, Gut microecology

## Abstract

**Background:**

Poultry is the major reservoir of *Campylobacter* that contributes to human campylobacteriosis and threatens food safety. Litter contact has been linked to *Campylobacter* colonization, but the gut microecological impact underlying this link remains not fully clear. Here, we sought to investigate the impact of the gut microecology on the presence of *Campylobacter* by examining the microbiota in the duodenum, jejunum, ileum, ceca, and feces from chickens raised on commercial litter and in individual cages at 0–57 days of age.

**Results:**

Through litter contact, the presence of *Campylobacter* was found to benefit from microecological competition among *Lactobacillus*, *Helicobacter*, and genera that are halotolerant and aerobic or facultatively anaerobic in the upper intestine, such as *Corynebacterium* and *Brachybacterium*. The presence was also promoted by the increased abundance in obligate anaerobic fermentation microbes, especially members of the orders *Clostridiales* and *Bacteroidales*. The longitudinal analysis supported the vertical or pseudo-vertical transmission but suggested that colonization might occur immensely at 7–28 days of age. We observed a host genetic effect on the gut microecology, which might lead to increased heterogeneity of the microecological impact on *Campylobacter* colonization.

**Conclusions:**

The findings advance the understanding of the gut microecological impact on *Campylobacter* presence in the chicken gut under conditions of litter contact and suggest that manipulations of the gut microecology, as well as the microbes identified in the *Campylobacter* association networks, might be important for the development of intervention strategies.

**Supplementary Information:**

The online version contains supplementary material available at 10.1186/s12866-021-02353-5.

## Background

Despite considerable global efforts, campylobacteriosis is still one of the most commonly reported foodborne infections in both developed [1, 2] and developing countries [3]. Poultry is known as the major reservoir of *Campylobacter* and the consumption of contaminated chicken meat is considered the main cause of human campylobacteriosis [1]. Therefore, on-farm control is important in campylobacteriosis intervention. To reduce *Campylobacter* in the chicken gut with the aim of diminishing the burden of campylobacteriosis, strict hygiene measures and biosecurity [4], and different feed additives, such as organic acids [4] and probiotics [5], have been used. Other potential strategies, such as bacteriophages [5], vaccines [6] and anti-*Campylobacter* bacteriocins [7], have also been proposed. However, *Campylobacter* colonization and prevalence are not yet well controlled [1].

The production environment is found to have impacts on *Campylobacter* colonization and prevalence. In an early study, higher isolation rates of *Campylobacter jejuni* were observed in broilers raised on litter than in those raised in cages [8]. While litter conditions have been reported to have no effect on *Campylobacter* colonization [9], studies have shown that chickens can be colonized by *Campylobacter* from contaminated litter through the fecal-oral route [10, 11]. These results indicate that contact between chickens and the litter would promote the development of *Campylobacter* colonization and its prevalence. Since *Campylobacter* survives within a complex gut microbial ecosystem instead of existing alone in the chicken gut, the interactions among microbes in the gut microecology should play an important role in *Campylobacter* colonization. However, how the gut microecology impacts the presence of *Campylobacter* in the chicken gut when in contact with litter remains not fully clear.

Increasing discoveries and perspectives on the gut microbiome have been reported from an ecological view [12–14], which requires as many taxa as possible to be examined in one study. Many previous studies on *Campylobacter* could examine only one or a few microorganisms with culture-dependent or low-throughput technologies [8, 15–17], which limited the identification of ecological relationships among microbes. Culture-independent high-throughput sequencing tools have brought new insights into understanding of microbial ecosystems. Through the use of high throughput sequencing technologies, chickens with high *Campylobacter* loads were observed to have increased gut microbial diversity [18–20] and an increased abundance of microbes, such as *Clostridium* [19, 21, 22] and *Lachnospiraceae* [19, 23]. However, the samples used in these studies were only from the ileum, ceca, or feces. While *Campylobacter* is enriched in the lower intestine instead of in the duodenum or jejunum, the interaction among microbes in the upper intestine might have an impact on the microbial composition as well as *Campylobacter* colonization in the lower intestine. Therefore, a comprehensive gut microecology covering the microflora from the upper and lower intestine as well as the feces is required to understand the role of the gut microecology on *Campylobacter* presence.

Most investigations and strategies proposed have focused on horizontal transmission of *Campylobacter*, and some studies have reported the lack of evidence for vertical transmission [15, 24, 25]. While studies have observed *Campylobacter jejuni* penetration through eggshells [26] and colonization in the egg contents [27], the vertical or pseudo-vertical transmission of *Campylobacter* has received little attention [27]. Thus, further study is needed to better understand the vertical or pseudo-vertical transmission of *Campylobacter* in chickens.

Host genetics have been largely observed to have impacts on the composition of the gut microbiota in humans [28–31]. A recent study revealed the role of the host’s genetics in manipulating fat deposition in chickens [32]. Therefore, the host genetic effect should be considered when investigating the gut microecology underlying the presence of *Campylobacte*r resulting from litter contact.

Here, we took advantage of high-throughput sequencing technology to identify the gut microecological impact on *Campylobacte*r presence in the chicken gut by comparing the gut microbiota from chickens raised on commercial litter with that in chickens raised in individual cages. Parents with pedigrees from a *Campylobacter*-positive population were used to generate the experimental chicks. We evenly allocated full and half-sib chicks from each family to two groups so that the chicks in the two groups had the same genetic and hatching environment background. The gut spatial and longitudinal analysis of the microbiota showed that *Campylobacter* colonization might benefit from microecological competition among *Lactobacillus*, *Helicobacter*, and genera that are halotolerant and aerobic or facultatively anaerobic and might be promoted by an increased abundance of obligate anaerobic fermentation microbes, especially members within the orders *Clostridiales* and *Bacteroidales*. *Campylobacter* colonization might commence at or before hatching, but *Campylobacter* could hardly colonize the chicks’ gut in the first week of age and may immensely colonize from 7 to 28 days of age. In addition, *Campylobacter* colonization is likely to be influenced by host genetics through the impact on the gut microecological composition.

## Results

### Longitudinal and gut spatial dynamics of the *Campylobacter* abundance

To investigate the microecological networks promoting *Campylobacter* colonization in the chicken gut, we examined the gut microbiota of chickens raised on commercial litter and used the gut microbiota of chickens raised in individual cages that are able to avoid fecal contact and cross contamination as the comparison group (Supplementary Table 1). The *Campylobacter*-positive population was used as the parent generation to generate the experimental chicks. Full- and half-sib chicks from each family were evenly allocated to two groups so that the subjects in the two groups shared the same genetic and hatching environment background.

**Fig. 1:**
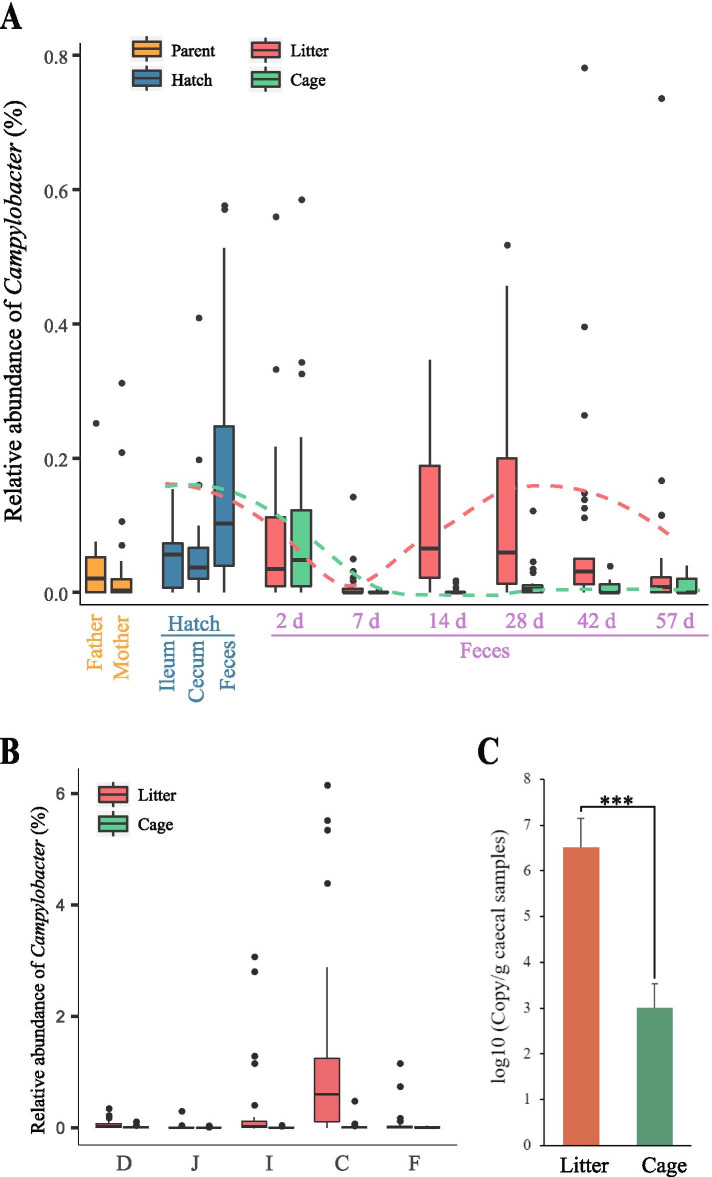
Longitudinal and gut spatial dynamics of the *Campylobacter* abundance. **A:** Relative abundance of *Campylobacter* in the litter and cage groups at different days of age as well as the *Campylobacter* abundance in parents’ fecal samples. **B:** Relative abundance of *Campylobacter* in the litter and cage groups in different gut sites at 57 days of age. D, J, I, C, and F denote the duodenum, jejunum, ileum, cecum, and feces, respectively. **C:** Quantitative changes in *Campylobacter* spp. in cecal samples by qPCR. One-way ANOVA was used to examine the significant difference between groups. ***P < 0.001

To characterize the *Campylobacter* presence, we examined the *Campylobacter* abundance using 16S rRNA gene sequencing with samples from 0 to 57 days of age in the two groups (Fig. 1A). Most chick samples at hatch were identified as having *Campylobacter* presence, including 98.3% of meconium, 76.9% of ileal and 96.2% of cecal samples. The *Campylobacter* abundance in both groups decreased from 0 to 7 days of age. At 7 days of age, *Campylobacter* was not detected in 70.0% of subjects in the litter group and in 96.7% in the cage group. However, the *Campylobacter* abundance in the litter group increased from 7 to 28 days of age and slowly decreased from 42 to 57 days of age, while no significant increase in abundance was observed in the cage group from 7 to 57 days of age. The longitudinal analysis suggests that *Campylobacter* could be carried by chicks though vertical/pseudo-vertical transmission, but *Campylobacter* could hardly colonize the chick gut or would be excreted out during the first week of life regardless of whether the chicks contacted the litter and suggests that intense colonization may occur from 7 to 28 days of age.

To understand the abundance of *Campylobacter* in both the upper and lower intestines in the two groups, we examined the *Campylobacter* abundance in the duodenum, jejunum, ileum, and feces (Fig. 1B). As expected, higher *Campylobacter* abundance was detected in the lower intestine than in the other sites. Since ceca are frequently examined in *Campylobacter* studies, we performed real-time quantitative PCR for cecal *Campylobacter* spp. and observed similar results as those from the high-throughput sequencing in which the *Campylobacter* abundance in the litter group was significantly higher than that in the cage group (Fig. 1C), suggesting that litter contact may promote the presence of *Campylobacter* in the chicken gut. Moreover, the comparative analysis suggests that the gut microbial contrast between chickens raised on conventional litter and those in individual cages is likely to be a good model to study *Campylobacter* colonization.

### Characterization of the gut microflora and its relationship with *Campylobacter* dynamics

**Fig. 2:**
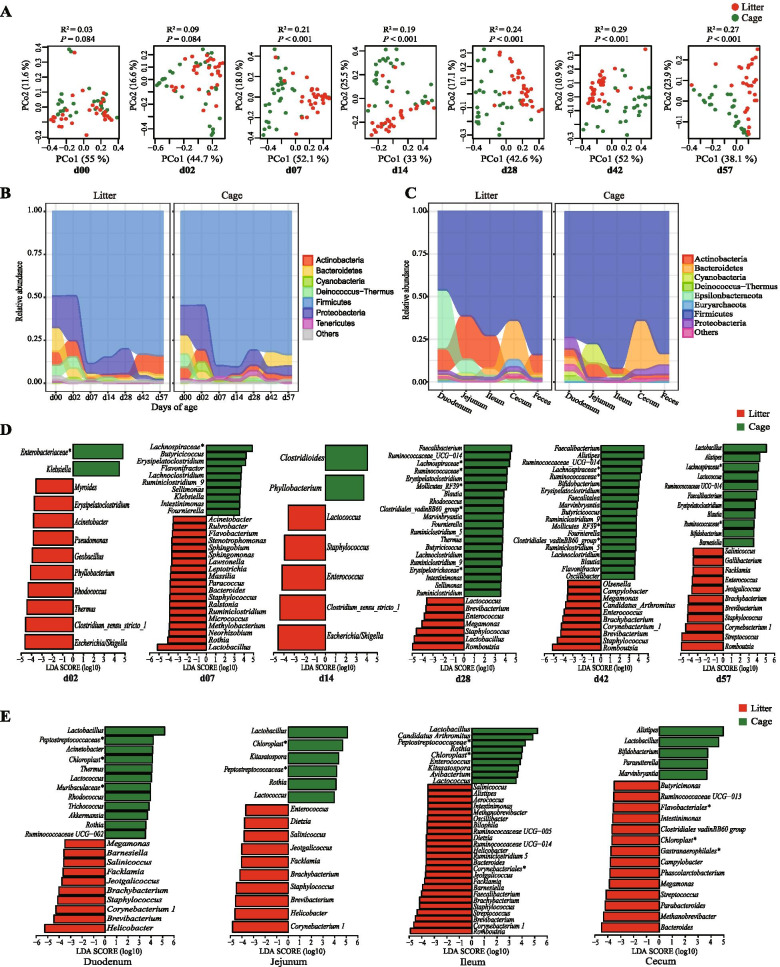
Longitudinal and gut spatial characterization of the gut microbiota. **A:** Principal coordinates analysis (PCoA) of the fecal microflora based on the weighted UniFrac distance in the litter and cage groups at different days of age. R,^2^ which is calculated from PERMANOVA, indicates the variation in distances explained by the grouping. **B:** Abundant phyla in litter and cage groups at different days of age. The average abundance for each of the phyla from samples at the corresponding days of age in each group was used to plot the alluvial diagram. **C:** Abundant phyla in the litter and cage groups at different gut sites. The average abundance of each of the phyla from samples at the corresponding days of age in each group was used to plot the alluvial diagram. **D:** Differential abundances of genera between the litter and cage groups at different days of age. **E:** Differential abundances of genera between the litter and cage groups at different gut sites. The nonparametric factorial Kruskal-Wallis (KW) sum-rank test was performed to detect significantly differential genera between the litter and cage groups at different days of age or at different gut sites. Linear discriminant analysis (LDA) scores were calculated for significantly different genera and then the scores were transformed by logarithm. Only genera with LDA scores over 3.5 are shown

To investigate the impact of gut microecology on the longitudinal dynamics of the *Campylobacter* abundance, we examined changes in gut microbial community over time in the litter and cage groups. The gut microbial community in the two groups significantly differentiated starting from 7 days of age (Fig. 2A), while the abundant microbes at the phylum (Fig. 2B) or genus (Supplementary Fig. 1) level varied between the two groups from as early as 2 days of age. Although both groups were dominated by Firmicutes in the whole experimental period, other abundant phyla varied over time in each group. Notably, the phylum Actinobacateria increased in abundance to become an abundant phylum in the litter group from 42 days of age (Fig. 2B). The increased abundance of this phylum was mainly due to genera that were found to be halotolerant and aerobic or facultative anaerobic, such as *Brachybacterium*, *Brevibacterium*, and *Corynebacterium*_1 (Supplementary Fig. 1). Except for *Lactobacillus* that dominated the gut microflora in both groups from 7 days of age, the majority of abundant microbes varied in taxonomy and abundance between the two groups. For example, the genera *Staphylococcus* and *Streptococcus* as well as the halotolerant and aerobic or facultative anaerobic genera, such as *Brachybacterium*, *Brevibacterium*, *Corynebacterium*_1, *Romboutsia*, *Facklamia*, Jeotgalicoccus, and Salinicoccus, were more abundant in the litter group, while microbes, such as *Faecalibacterium*, uncharacterized *Ruminococcaceae* genus, *Ruminococcaceae* UCG − 014, uncharacterized *Lachnospiraceae* genus, and *Alistipes* were more abundant in the cage group at the corresponding days of age (log10(LDA score) > 3.5; Fig. 2D and Supplementary Fig. 1). The results suggest that the increased abundances of the phylum Actinobacateria and the genera that are halotolerant and aerobic or facultative anaerobic largely contribute to the microecological differences and might contribute to the *Campylobacter* presence.

We examined the abundant and significantly differential microbes in samples from different gut sites to investigate the impact of the gut spatial microflora on *Campylobacter* dynamics. The phylum Firmicutes and genus *Lactobacillus* were found to be the dominant taxa at all examined gut sites in both the litter and cage groups at the phylum and genus levels, respectively (Fig. 2C; Supplementary Fig. 2). The genus *Helicobacter*, sharing a similar taxonomic lineage with *Campylobacter* within the phylum Epsilobacteraeota and order *Campylobacterales*, was found to be one of the most abundant microbes of the upper intestinal microflora in the litter group and was significantly higher in abundance than that in the cage group (log10(LDA score) > 3.5; Fig. 2E). In addition, the abovementioned halotolerant and aerobic or facultative anaerobic genera were also more abundant in the upper intestine in the litter group than in the cage group (log10(LDA score) > 3.5; Fig. 2E). Although the cecal microflora in both groups were dominated by similar microbes, such as *Alistipes*, *Faecalibacterium*, *Lactobacillus*, *Ruminococcaceae* UCG-14, uncharacterized *Lachnospiraceae* genus, and uncharacterized *Clostridiales* vadin BB60 group, the abundances of these microbes were significantly different between the two groups (log10(LDA score) > 3.5; Fig. 2E). Within these microbes, such as *Bacteroides*, *Barnesiella*, *Butyricimonas*, *Fecalibacterium*, *Oscillibacter*, *Phascolarctobacterium*, and *Megamonas*, obligate anaerobic fermentation microbes were found to be more abundant in the hindgut of the litter group than in the cage group (log10(LDA score) > 3.5).

**Fig. 3:**
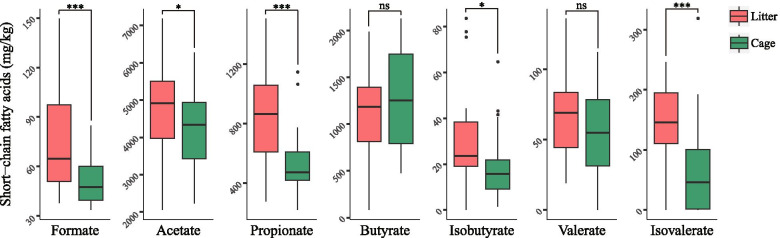
Comparisons of cecal short-chain fatty acids between the litter and cage groups. One-way ANOVA was used to examine the significant difference between groups. ***P < 0.001, *P < 0.05, and ns means no significance

Most of these significantly differential microbes in ceca were short-chain fatty acid (SCFA)-producing bacteria and were mainly from the orders *Bacteroidales* and *Clostridiales*. To ensure that these bacteria were actively producing SCFAs in the hindgut, we measured the cecal SCFAs, including formate, acetate, propionate, butyrate, isobutyrate, valerate, and isovalerate, and found that all measured SCFAs were significantly or marginally higher in the litter group than in the cage group (*P* < 0.05; acetate: *P* < 0.1), except for butyrate and valerate (*P* > 0.1) (Fig. 3). Higher SCFAs levels support the enrichment of SCFA-producing bacteria and more SCFA-producing activities in the hindgut of the litter group. Moreover, higher SCFAs might be linked to the higher abundance of *Campylobacter,* as studies found that *Campylobacter* may benefit from the anaerobic fermentation by consuming SCFAs [33, 34].

Therefore, both compositional and differential analysis suggest that *Helicobacter* and genera that are halotolerant and aerobic or facultative anaerobic in the small intestine as well as obligate anaerobic fermentation microbes in the lower intestine might contribute to the *Campylobacter* presence in the chicken gut.

### Gut microecological networks underlying *Campylobacter* presence

**Fig. 4:**
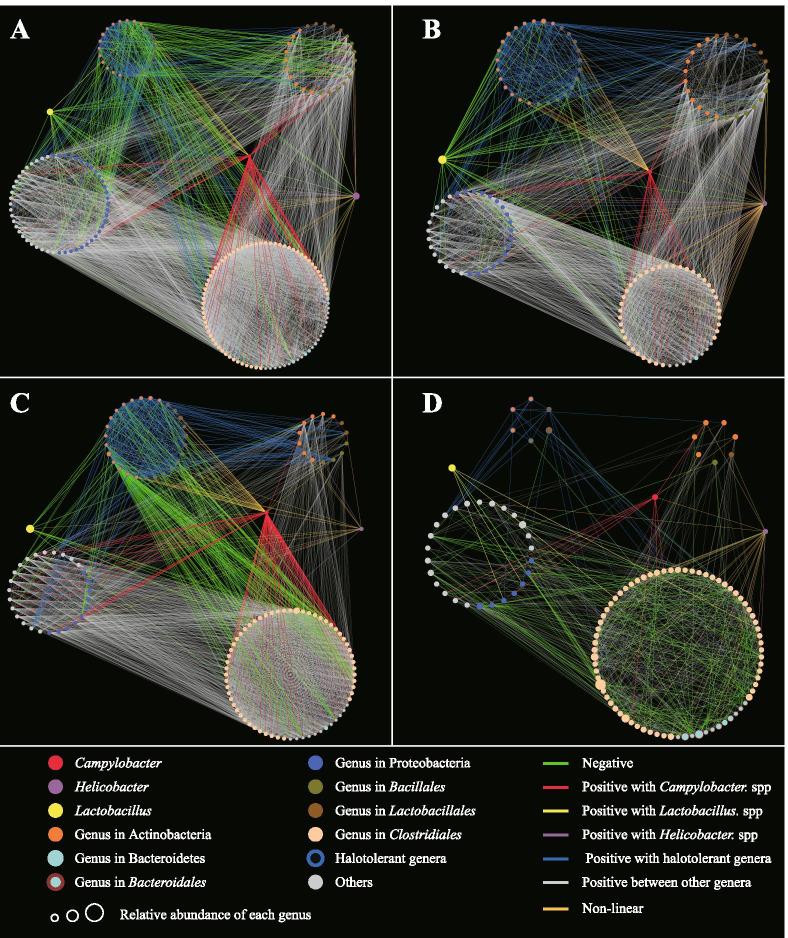
*Campylobacter* associated microecological networks in the duodenum (**A**), jejunum (**B**), ileum (**C**), and ceca (**D**). Each dot denotes one genus in the corresponding intestinal site. Different taxa are filled with different colors. The dots filled with red, purple, and yellow represent *Campylobacter*, *Helicobacter*, and *Lactobacillus*, respectively. The dot with a blue circle denotes genera that are halotolerant and aerobic or facultative anaerobic. Positive associations between *Campylobacter* and other microbes are connected by red lines. Negative associations between microbes are connected with green lines. The genera with close taxonomy are grouped together. The size of each dot denotes the abundance of the corresponding genus; that is, the larger the dot size is, the higher the genus abundance is. Halotolerant genera denote genera that are halotolerant and aerobic or facultative anaerobic. Only significant correlations (*P* < 0.05) over 0.3 or lower than − 0.25 are shown

To further characterize how the gut microecology promotes the presence of *Campylobacter*, we constructed microbial networks based on correlation analysis for the microbes at each of the examined gut sites (Fig. 4). In total, 115 genera, including 114 bacteria and 1 archaeon (genus *Methanobrevibacter*), showed relationships with *Campylobacter* (Supplementary Fig. 3). However, most of the identified relationships were positive while only three bacteria (*Corynebacterium*_1, *Dietzia*, and *Staphylococcus*) showed negative associations with *Campylobacter* (Fig. 4A, C and Supplementary Fig. 3). All three bacteria were found to be halotolerant and aerobic or facultative anaerobic genera. Other bacteria associated with *Campylobacter* were mainly from the orders *Clostridiales* and *Bacteroidales*. Of these bacteria, 53 were from *Clostridiales* (30 in *Ruminococcaceae*, 15 in *Lachnospiraceae* and 8 in others) and 14 were from *Bacteroidales* (3 in *Prevotellaceae*, 3 in *Rikenellaceae*, 3 in *Barnesiellaceae*, 2 in *Marinifilaceae*, and 3 in others) (Fig. 4, Supplementary Fig. 3, and Supplementary Table 2). The abundance of these bacteria accounted for 60.4% of the genera associated with *Campylobacter*. Interestingly, although *Campylobacter* was found to be enriched in the ceca, it was positively associated with more microbes in the small intestine (63, 24, and 69 microbes in the duodenum, jejunum, and ileum, respectively) than in the ceca (8 microbes) (Fig. 4 and Supplementary Fig. 3). In addition, seven microbes (*Barnesiella*, *Lachnospiraceae*_UCG-010, *Fournierella*, *Oscillibacter*, *Ruminiclostridium*_9, *Ruminococcaceae*_UCG-007, and *Megamonas*) were positively associated with *Campylobacter* in all examined small intestinal sites with five of them belonging to the order *Clostridiales*. These results suggest that obligate anaerobic fermentation microbes, especially members within the order *Clostridiales* in both the upper and lower intestines, were associated with the *Campylobacter* presence.

**Fig. 5:**
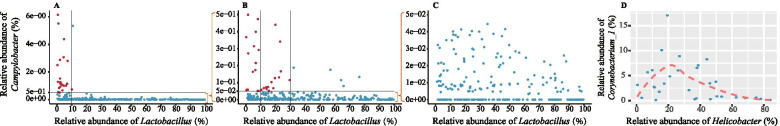
Nonlinear relationships between microbes. The relationship between *Campylobacter* and *Lactobacillus* in duodenal, jejunal, ileal, cecal, and fecal samples at 57 days of age where the *Campylobacter* abundance was: ≥ 0 (**A**), ≥ 0 and ≤ 0.5% (**B**), and ≥ 0 and ≤ 0.05% (**C**). **D** The nonlinear relationship between *Helicobacter* and *Corynebacterium*_1 in the duodenum

Nonlinear microecological relationships appeared to play important roles in *Campylobacter* colonization as well. *Lactobacillus* and many halotolerant and aerobic or facultative anaerobic genera showed negative relationships with *Campylobacter*, but the relationships were not linear (Fig. 5). For example, when the abundance of *Lactobacillus* was over 10%, the *Campylobacter* abundance was lower than 0.5% in most samples (Fig. 5A). When *Lactobacillus* abundance was over 30%, the *Campylobacter* abundance in most samples was lower than 0.05% (Fig. 5B). Although *Campylobacter* still showed a negative relationship trend with *Lactobacillus* when the *Campylobacter* abundance was lower than 0.05%, the susceptibility of *Campylobacter* to *Lactobacillus* decreased (Fig. 5C). This result suggests that *Lactobacillus* has good potential to largely suppress *Campylobacter* colonization but could not thoroughly clear it. Similar results were also observed between *Campylobacter* and the halotolerant and aerobic or facultative anaerobic genera in the small intestine (Supplementary Fig. 4), which suggested that the halotolerant and aerobic or facultative anaerobic genera might also play an important role in the disruption of *Campylobacter* colonization.

However, the increase in *Helicobacter* is likely to mitigate the disruption of *Campylobacter*. *Helicobacter* showed a negative relationship with *Lactobacillus* (Fig. 4 and Supplementary Table 3), which might ameliorate the suppression of *Campylobacter* with the increase in *Helicobacter* abundance. Moreover, nonlinear relationships were observed between *Helicobacter* and genera that are halotolerant and aerobic or facultative anaerobic (Fig. 5D and Supplementary Fig. 5). The relationships between these microbes were found to shift from positive to negative with increasing *Helicobacter* abundance. For instance, *Helicobacter* showed a synergistic relationship with *Corynebacterium*_1 when *Helicobacter* abundance was lower than 30%, but the relationship shifted to become antagonistic when the abundance of *Helicobacter* was over 30%. The abundance of *Corynebacterium*_1 even decreased to close to zero when the abundance of *Helicobacter* was over 50%. Notably, the genera that are halotolerant and aerobic or facultative anaerobic also showed negative relationships with *Lactobacillus* (Fig. 4 and Supplementary Table 3), which might further mitigate the disruption of *Campylobacter* colonization. The results suggest that although *Lactobacillus* and genera that are halotolerant and aerobic or facultative anaerobic might disrupt *Campylobacter* colonization in the chicken gut, the microecological competition among these microbes and *Helicobacter* might mitigate the disruption.

### Impact of litter contact and host genetics on the gut microecological uniformity

**Fig. 6:**
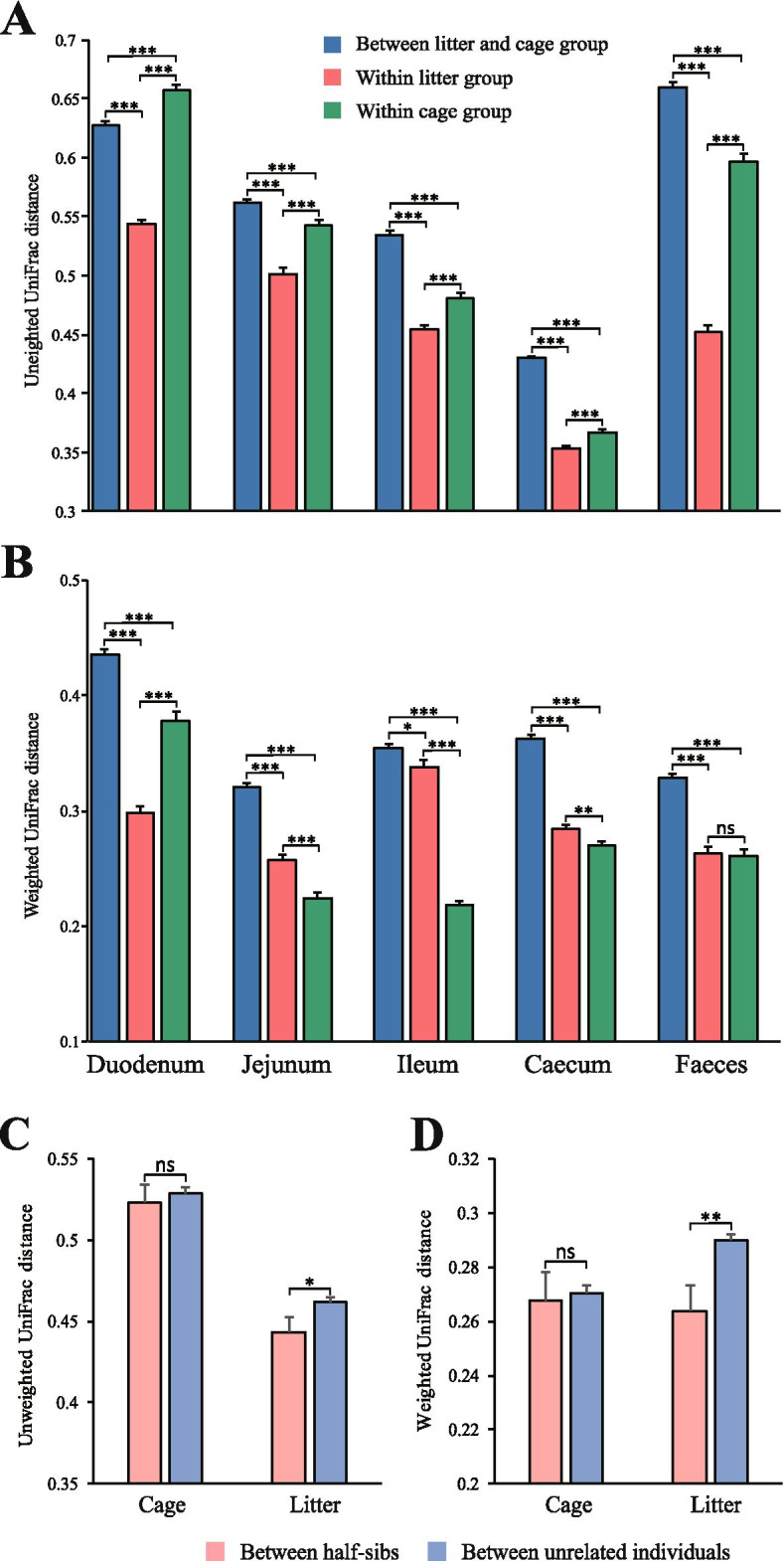
UniFrac distance based microbial community dissimilarity in different groups. Unweighted (**A**) and weighted (**B**) UniFrac distances within the litter or cage group at different gut sites. Unweighted (**C**) and weighted (**D**) UniFrac distances within half sibs or unrelated individuals at different gut sites. One-way ANOVA was used to examine the significant difference between groups. ***P < 0.001, **P < 0.01, *P < 0.05, and ns means no significance

We next examined the microbial similarity within each group based on interindividual UniFrac distances to investigate to what extent litter contact and host’s genetics could affect the uniformity of the microbial community, which might subsequently influence the spread of *Campylobacter* in the population (Fig. 6). The average unweighted UniFrac distance was found to be lower in the litter group than in the cage group at all examined gut sites (*P* < 0.001; Fig. 6A), indicating that litter contact increases the uniformity of microbial species in both the upper and lower intestines as well as in the feces. Nevertheless, while the average weighted UniFrac distance in the litter group was consistently lower than that in the cage group in the duodenum, the distance was higher in the litter group in the jejunum, ileum, and ceca (*P* < 0.05; Fig. 6B). This means that after increasing the microbial species uniformity, the variation in microbial abundance was increased. Since the environment and diet were well controlled, the major factor might be the host’s genetics, which might lead to increased microbial abundance variation [32]. We examined the interindividual UniFrac distance based on the genetic relationship and found that the distance among half sibs was lower than that among unrelated individuals regardless of whether only the microbial species were considered (Fig. 6C) or if the microbial abundance was taken into account (Fig. 6D). Moreover, such a trend was observed in the litter group but not in the cage group, which supported that the increased microbial species uniformity and the host genetic heterogeneity might contribute to the increased microbial abundance variation. The results suggest that litter contact largely contributes to the uniformity of microbial species, but the host genetic effect on the gut microecology might lead to increased heterogeneity of the microecological impact on the presence of *Campylobacter*.

## Discussion

In this study, we investigated the impact of the gut microecology on *Campylobacter* presence by comparing the gut microbiota of chickens raised on the litter floor from 0 to 57 days of age with the gut microbiota from chickens raised in individual cages. The results revealed that the presence of *Campylobacter* in the chicken gut might (a) benefit from microecological competition among *Lactobacillus*, *Helicobacter*, and genera that are halotolerant and aerobic or facultative anaerobic in the upper intestine; (b) be promoted by the increased abundance of obligate anaerobic fermentation microbes in the gut, especially members within the orders *Clostridiales* and *Bacteroidales*; (c) occur immensely during 7–28 days of age; and (d) be influenced by host genetics through the impact on the gut microecological composition.

The use of individually caged birds as the comparison group facilitated a better observation of the impact of litter contact on the gut microecology and its association with the presence of *Campylobacter* in the conventional production environment within a relatively natural habitat setting. Moreover, this contrast facilitated a better understanding of the different contributions of litter contact and host genetics to the gut microecological structure. The result in this study that caged birds were observed to have a significantly lower *Campylobacter* abundance is consistent with that in the previous study [8]. Compared to the previous study [8], the use of high-throughput sequencing technology and examination of the different gut sites instead of only the feces provided an opportunity to observe the gut microecology underlying the difference in *Campylobacter* abundance between the two groups.

The microecology in the upper intestine has rarely been reported in previous studies that investigated *Campylobacter* colonization in chickens. In the current study, the presence of *Campylobacter* was linked to many obligate anaerobes within the orders *Clostridiales* and *Bacteroidales* in the ileum or ceca, which agrees with the observations in previous studies [19, 21, 35]. Moreover, this study found that the *Campylobacter* presence showed more positive associations with these obligate anaerobic fermentation microbes in the small intestine than with those in the ceca. This might be because *Campylobacter* is always mucosally adherent in the ceca [33], and its proliferation is likely to be limited by the mucosal surface area, which leads to less linear relationships with these anaerobes in the ceca, although *Campylobacter* might benefit from anaerobic fermentation by consuming SCFAs [33].

In line with the findings in this study, *Lactobacillus* has been previously reported to have an inhibitory effect on *Campylobacter* [36, 37]. Therefore, many *Lactobacillus*-related probiotic products have been proposed to prevent or reduce the prevalence of *Campylobacter* [36, 37]. Nevertheless, the results of the current study found that *Lactobacillus* might not thoroughly clear *Campylobacter*. Moreover, redundant *Lactobacillus* might suppress most other microorganisms, including some beneficial microorganisms (Supplementary Table 3), and subsequently reduce the diversity of the gut microbial community and disrupt the microecological balance (Supplementary Fig. 6), which agrees with the previous observation [38]. Thus, although *Lactobacillus* is effective in controlling *Campylobacter*, it should be added with caution.

The increased abundance of genera that are halotolerant and aerobic or facultative anaerobic in the small intestine may play an important role in promoting *Campylobacter* colonization, which was also rarely reported in previous studies. Most of these microbes are abundant taxa in the litter microflora as well (Supplementary Table 4), which agrees with previous observations [10, 39]. These microbes showed negative associations with *Lactobacillus* (Supplementary Table 3). This might facilitate the colonization of microaerophilic and acid-tolerant *Helicobacter* in duodenum [10, 39], as *Helicobacter* showed negative relationships with *Lactobacillus* and nonlinear relationships with genera that are halotolerant and aerobic or facultative anaerobic (Supplementary Table 4 and Supplementary Fig. 5). Therefore, competitions among these microbes might mitigate the disruption of *Lactobacillus* to *Campylobacter* in the chicken gut.

Positive detection of *Campylobacter* in the gut of newly hatched chicks supports vertical or pseudo-vertical transmission. Some previous studies have reported the negative detection of *Campylobacte*r during the first 1 or 2 weeks post-hatching [15, 40], known as the lag phase [15, 41]. However, *Campylobacter* was detected in this study in intestinal and fecal samples from posthatching chicks that originated from a *Campylobacter*-positive parental population. In line with this finding, *Campylobacter* has been shown to penetrate eggshells [26] and egg contents through oviduct colonization and fecal contamination [26]. Similarly, some studies have detected *Campylobacter* in the gut of embryos [42], newly hatched chicks and hatchery fluff [43]. Therefore, the development of *Campylobacter* colonization might commence at or before hatching, but *Campylobacter* could hardly colonize the chicks’ gut in the first week of life and may immensely colonize from 7 to 28 days of age, which is similar to the results reported in previous studies [15, 35, 44]. This suggests that strategies to prevent vertical or pseudo-vertical transmission should be given attention in farm production.

There are several limitations to the present study. First, the use of solely the V4 region of the 16S rRNA gene in this study might limit the identification of taxonomy at the species level compared to the joint use of regions, such as V3-V5 and V6-V9 [45]. Moreover, a study showed that the use of V2-V3 regions demonstrated higher resolution in taxonomy identification at the genus and species levels than the use of the V3-V4 regions [46]. These results suggest that some species might not be distinguished because of identification limitations in taxonomy at the species level using only the V4 region. However, while some subregion combinations may perform better than others, different subregions would have bias in identifying bacterial taxa [45]. As such, to better understand the taxonomy at a lower-rank level, such as the species or even the strain level, the use of full V1–V9 regions or metagenomics might be more appropriate. Second, although we examined chicks’ microbiota from as early as the hatch day and sampled at seven time points, we might miss some information regarding the alteration of gut microbiota as there are gaps between two time points. Therefore, a longitudinal day to day examination might be required in future studies that could help to reveal more accurate changes in the gut microbiota [35]. Moreover, environmental pressure has been found to have a significant impact on the microbial community structure significantly and has been linked to *Campylobacter* presence [20]; therefore, factors such as stocking intensity should be considered in future studies when investigating the gut microflora and *Campylobacter* colonization in the chicken gut.

## Methods

### Subjects, housing, and sample collection

The complete procedure was performed according to the guidelines established by the Animal Care and Use Committee of China Agricultural University (Permit Number: AW08059102–1).

A pure line of slow-growing yellow broilers was used in this study. The birds were obtained from Jiangsu Lihua Poultry Breeding Co., Ltd. in Jiangsu Province, China. A pure line of broilers with *Campylobacter* detection positivity and clearly recorded kinship was selected. Twelve families were established as the parent generation, and each family consisted of one male and nine females. The birds were kept in individual cages, and artificial insemination was performed to maintain the mating balance and efficiency. At 36 weeks of age, the fertile eggs from the parent generation were collected and incubated. The incubation was performed under the standard incubation procedure, including a strict incubation period, temperature, humidity, and sterilization. After hatching, three full-sib male chicks from each mother were selected and randomly allocated into three groups. One group was used for ileal and cecal sampling, and the other two were retained for the subsequent experiment. One of the retained groups was conventionally raised in the same pen on the floor covered with fresh rice hulls as the litter, and the other group was kept in individual cages to avoid litter contact and cross contamination and served as the comparison group. The bottom of the cage was mesh to allow the feces to drop through, which avoided fecal contact. Both groups were fed with the same diet. To reduce the influences of other environmental factors, the housing conditions were similarly maintained in the two groups according to the housing standards. Since drugs, prebiotics, probiotics, and antibiotics may intensively affect the composition of the gut microbiota [47–49], none of them were used during the experimental period.

Fecal samples were collected from the parents when completing the collection of fertile eggs. We collected meconium from the retained groups and the ileal and cecal mucosal surfaces from the other group after hatching. Fecal samples were collected once the excreta were discharged at 2, 7, 14, 28, 42, and 57 days of age (Supplementary Table 1). The middle of the feces was collected to avoid environmental contamination. Ten families were randomly selected, and three full-sib pairs in each family were randomly selected for the following intestinal sampling. At 57 days of age, these birds were humanely euthanized by cervical dislocation and subsequently dissected. The contents and mucosal surfaces of the duodenum, jejunum, ileum, and ceca were collected immediately after dissection. To ensure the uniformity of samples among individuals, a 10-cm-long fixed section of the duodenum and jejunum, the whole ileum and a pair of ceca were selected for sampling. Therefore, samples from 30 chickens in each group were used for further analysis. In total, there were 330 samples from each group (Supplementary Table 1). The contents and mucosa were mixed well before collection. All samples were immediately frozen in liquid nitrogen and then stored at − 80 °C. Both the intestinal contents and mucosa were sampled, since the microbes from both sources may contribute to host interactions with respect to nutrient metabolism and immunity [50]. Fresh litter samples were collected when the litter was put into the experimental pen. Litter and trough water samples were also collected. Feed samples were collected at the beginning and end of the experiment.

### DNA extraction and 16S rRNA gene sequencing

Total DNA was extracted from intestinal and fecal samples using the OMEGA E.Z.N.A. Stool DNA Kit (#D4015) following the manufacturer’s instructions. The V4 region (515F-806R) of the 16S rRNA gene was employed to generate indexed libraries for sequencing. All PCRs were carried out in 30 μL reactions with 15 μL of Phusion® High-Fidelity PCR Master Mix (New England Biolabs), 0.2 μM forward and reverse primers, and approximately 10 ng template DNA. Thermal cycling consisted of an initial denaturation at 98 °C for 1 min, followed by 30 cycles of denaturation at 98 °C for 10 s, annealing at 50 °C for 30 s, and elongation at 72 °C for 30 s. Finally, 72 °C for 5 min. Mix the same volume of 1× loading buffer (contained SYB green) with PCR products and operate electrophoresis on 2% agarose gel for detection. PCR products were mixed in equidensity ratios. Then, mixture PCR products were purified with GeneJETTM Gel Extraction Kit (Thermo Scientific). Sequencing libraries were generated using Ion Plus Fragment Library Kit 48 rxns (Thermo Scientific) following manufacturer’s recommendations. The library quality was assessed on the Qubit@ 2.0 Fluorometer (Thermo Scientific). At last, the library was sequenced on an Ion S5TM XL platform and 400 bp single-end reads were generated. Single-end reads were assigned to samples based on their unique barcode and truncated by cutting off the barcode and primer sequence.

### Analysis of sequencing data

The sequence data were resolved to amplicon sequence variants (ASVs) instead of operational taxonomic units (OTUs). ASVs are considered to be a replacement for OTUs based on accumulated evidence and views, such as the improvement in accuracy, reusability, reproducibility and comprehensiveness [51–56]. Sequence quality control, feature table construction and taxonomic annotation were performed using DADA2 [51]. Specifically, sequences were filtered and trimmed to obtain high-quality data. The length of sequences to be trimmed was set as 234 bp to ensure that at least 9900 bases could be randomly sampled for quality evaluation at all retained positions. The median of the quality score was 28, and more than 75% of the bases were over 20 at position 234 bp. None of the bases was trimmed at the beginning position of the reads, as the quality score of more than 90% of bases at the first 15 bp positions was over 20. The parameters HOMOPOLYMER_GAP_PENALTY and BAND_SIZE were set as − 1 and 32, respectively, following the recommendation of the tutorial (https://benjjneb.github.io/dada2/faq.html). The singleton ASVs were discarded before removing chimeras because they were generated mainly by sequencing errors. The chimeras were removed using the “consensus” method, and the taxonomy was assigned with the SILVA132 database [57, 58]. Next, microbial diversity analysis was performed using QIIME [59] with a QIIME2 pipeline (https://qiime2.org). The results from DADA2 were transformed to the format required in QIIME2 and we rarefied the data to 0.9 × lowest numbers of sequences to control for sampling effort in diversity analysis.

The microbial community similarities among samples from different days of age or samples from different gut sites were compared by performing principal coordinates analysis (PCoA) with UniFrac distance. The results were tested for significance by PERMANOVA using *vegan* in R. The microbial community similarities within the litter or cage group were calculated with interindividual UniFrac distances. Similarly, the microbial community similarities were calculated for individuals with or without genetic relationships. One-way ANOVA was used to examine the significant differences in the UniFrac distance between two groups. The Shannon diversity index was calculated to examine the alpha diversity of the gut microbial community using *vegan* in R. We performed one-way ANOVA to compare the Shannon diversity index at each gut site between the two groups.

The *Campylobacter* abundances in samples at different days of age and different gut sites are shown with boxplots using ggplot2 in R. The average abundances of abundant phyla in the litter and cage groups at different days of age or different gut sites are shown with alluvial diagrams using geom_alluvium in R. The nonparametric factorial Kruskal-Wallis sum-rank test and linear discriminant analysis were performed in LEfSe [60] to identify the differentially abundant genera between the two groups. To decrease the data noise, only genera with an average relative abundance > 0.001 at each sampling site were used for LEfSe.

The associations among microorganisms were determined at the genus level with Pearson correlation analysis by psych in R at each sampling site. Nonlinear association fitting between *Helicobacter* and genera that are halotolerant and aerobic or facultative anaerobic was performed using geom_smooth in R with the loess method. Only the genera present in over 6 samples were used in the association analysis.

### Measurement of short-chain fatty acids

A 0.5 g cecal sample was weighed into a 10 ml polypropylene tube, and 8 ml deionized water was added. After using an ultrasonic bath for 30 min, the mixture was centrifuged for 10 min at 8000 rpm. The resulting suspension was diluted 10 times and filtered through a 0.22 μm filter. Then, 25 μL of extracted sample solution was analysed by high performance ion chromatography with ICS-3000 (Dionex, USA) and determined by conductivity detection. The organic acids were separated on an AS11 analytical column (250 mm × 4 mm) and an AG11 guard column under the following gradient conditions: the gradient was carried out with potassium hydroxide; 0–5 min, 0.8–1.5 mM; 5–10 min, 1.5–2.5 mM, and 10–15 min, 2.5 mM, and the flow rate was 1.0 ml/min. One-way ANOVA was performed to test if the SCFA was significantly different between two groups.

### Quantitative real-time PCR

Numbers of *Campylobacter* spp. (e.g. *C. jejuni*, *C. coli*, *C. lari*, and *C. hyointestinalis*) were quantified by real-time quantitative PCR (qPCR) using specific primers (forward primer: 5′- CACGTGCTACAATGGCATATACAA-3′; reverse primer: 5′- CCGAACTGGGACATATTTTATAGATTT-3′), generically targeting *Campylobacter* spp. 16S rDNA sequence according to de Boer et al. [61]. The reference strain was synthesized according to methods as previously described [62], and the 16S rRNA gene was cloned into a pMD-18 T Vector System. Real-time PCR was performed on an Applied Biosystems 7500 thermal cycler using Applied Biosystems® Power SYBR® Green PCR Master Mix. The real-time PCR assay was carried out in a 15 μl volume and contained 1 μl DNA, 7.5 μl Applied Biosystems® Power SYBR® Green PCR Master Mix, 0.3 μl (10 pmol) of forward and reverse primers and 5.9 μl DNase-free water. The cycling conditions consisted of 3 min at 95 °C, followed by 40 cycles of 30 s at 95 °C, 30 s at 60 °C and 40 s at 72 °C. The standards of *Campylobacter* spp. were diluted to yield a series of 10-fold concentrations and then used for standard curves. The number of copies of *Campylobacter* spp. was transformed by logarithm. The transformed data was used for a one-way ANOVA test to examine the significant difference between two groups.

## Supplementary Information

**Additional file 1: Supplementary Figure 1.** Average relative abundances of predominant genera in the litter and cage groups at different days of age. Only genera with average relative abundance over 2% are shown with taxonomic annotation. **Supplementary Figure 2.** Average relative abundances of predominant genera in the litter and cage groups in different gut sites at 57 days of age. Only genera with relative abundance over 2% are shown with taxonomic annotations. D, J, I, C, and F denote duodenum, jejunum, ileum, cecum, and feces, respectively. **Supplementary Figure 3.** Associated with Fig. 4. Heatmap of correlations between *Campylobacter* and microbes in different intestinal segments. Only significant correlations (*P* < 0.05) over 0.3 or below − 0.25 are shown. **Supplementary Figure 4.** Nonlinear relationships between *Campylobacte*r and genera that are halotolerant and aerobic or facultative anaerobic in the small intestine. **Supplementary Figure 5.** Nonlinear relationships between *Helicobacter* and genera that are halotolerant and aerobic or facultative anaerobic in duodenum. **Supplementary Figure 6.** The Shannon index between the litter and cage groups in different gut sites at 57 days of age.

**Additional file 2: Supplementary Table 1.** The chicken genetic family structure and samples of the progeny population. **Supplementary Table 2.** Summary of microbes associated with *Campylobacter*. **Supplementary Table 3.** Correlations between *Lactobacillus* and microbes at the genus level in different intestinal segments. **Supplementary Table 4.** The relative abundance of microorganisms in used litter.

## Data Availability

The raw data on which the conclusions of the manuscript rely have been deposited in the National Center for Biotechnology Information (NCBI) database (accession numbers: PRJNA542479).
