# Supplementary material for: Impact of the gut microecology on Campylobacter presence revealed by comparisons of the gut microbiota from chickens raised on litter or in individual cages
Source: BMC Microbiol. 2021 Oct 22;21:290. doi: 10.1186/s12866-021-02353-5 (PMC8532315; doi:10.1186/s12866-021-02353-5)
Supplement: Supplementary file 1 — Additional file 1: Supplementary Figure 1. Average relative abundances of predominant genera in the litter and cage groups at different days of age. Only genera with average relative abundance over 2% are shown with taxonomic annotation. Supplementary Figure 2. Average relative abundances of predominant genera in the litter and cage groups in different gut sites at 57 days of age. Only genera with relative abundance over 2% are shown with taxonomic annotations. D, J, I, C, and F denote duodenum, jejunum, ileum, cecum, and feces, respectively. Supplementary Figure 3. Associated with Fig. 4. Heatmap of correlations between Campylobacter and microbes in different intestinal segments. Only significant correlations (P < 0.05) over 0.3 or below − 0.25 are shown. Supplementary Figure 4. Nonlinear relationships between Campylobacter and genera that are halotolerant and aerobic or facultative anaerobic in the small intestine. Supplementary Figure 5. Nonlinear relationships between Helicobacter and genera that are halotolerant and aerobic or facultative anaerobic in duodenum. Supplementary Figure 6. The Shannon index between the litter and cage groups in different gut sites at 57 days of age. [file 12866_2021_2353_MOESM1_ESM.pdf]

Supplementary Information for

Impact of the gut microecology on *Campylobacter* presence revealed by comparisons of the gut microbiota from chickens raised on litter or in individual cages

Wei Yan[a, b], Qianqian Zhou[a, b], Zhongyang Yuan[a, b], Liang Fu[a, b], Chaoliang Wen[a, b], Ning Yang[a, b], Congjiao Sun[a, b, *]

[a] Poultry Science Laboratory, College of Animal Science and Technology, China Agricultural University, Beijing, 100193, China.

[b] National Engineering Laboratory for Animal Breeding and Key Laboratory of Animal Genetics, Breeding and Reproduction, Ministry of Agriculture and Rural Affairs, Beijing, 100193, China.

* Correspondence to: Congjiao Sun, cjsun@cau.edu.cn

Supplementary Fig. 1. Average relative abundances of predominant genera in the litter and cage groups at different days of age. Only genera with average relative abundance over 2% are shown with taxonomic annotation.

Supplementary Fig. 2. Average relative abundances of predominant genera in the litter and cage groups in different gut sites at 57 days of age. Only genera with relative abundance over 2% are shown with taxonomic annotations. D, J, I, C, and F denote duodenum, jejunum, ileum, cecum, and feces, respectively.

Supplementary Fig. 3. Associated with Fig. 4. Heatmap of correlations between *Campylobacter* and microbes in different intestinal segments. Only significant correlations ($P < 0.05$) over 0.3 or below -0.25 are shown.

Supplementary Fig. 4. Nonlinear relationships between *Campylobacte*r and the genera that are halotolerant and aerobic or facultative anaerobic in the small intestine.

Supplementary Fig. 5. Nonlinear relationships between *Helicobacter* and genera that are halotolerant and aerobic or facultative anaerobic in duodenum.

Supplementary Fig. 6. The Shannon index between the litter and cage groups in different gut sites at 57 days of age.

Supplementary Table 1. The chicken genetic family structure and samples of the progeny population.

Supplementary Table 2. Summary of microbes associated with *Campylobacter*.

Supplementary Table 3. Correlations between *Lactobacillus* and microbes at the genus level in different intestinal segments.

Supplementary Table 4. The relative abundance of microorganisms in used litter.

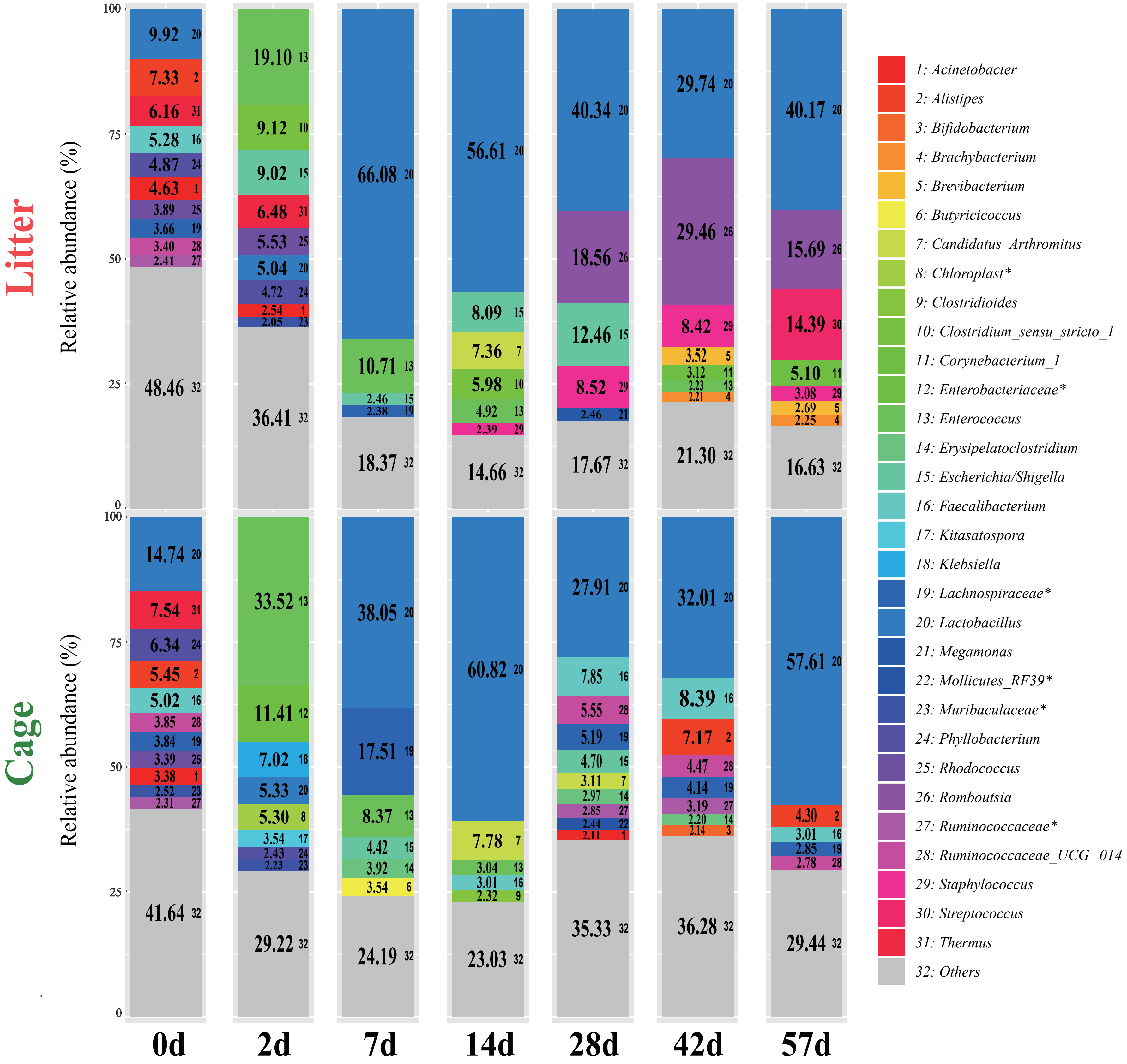

Supplementary Fig. 1: Average relative abundances of predominant genera in litter and cage groups at different days of age. Only the genera with relative abundances over 2% are shown with taxonomic annotations.

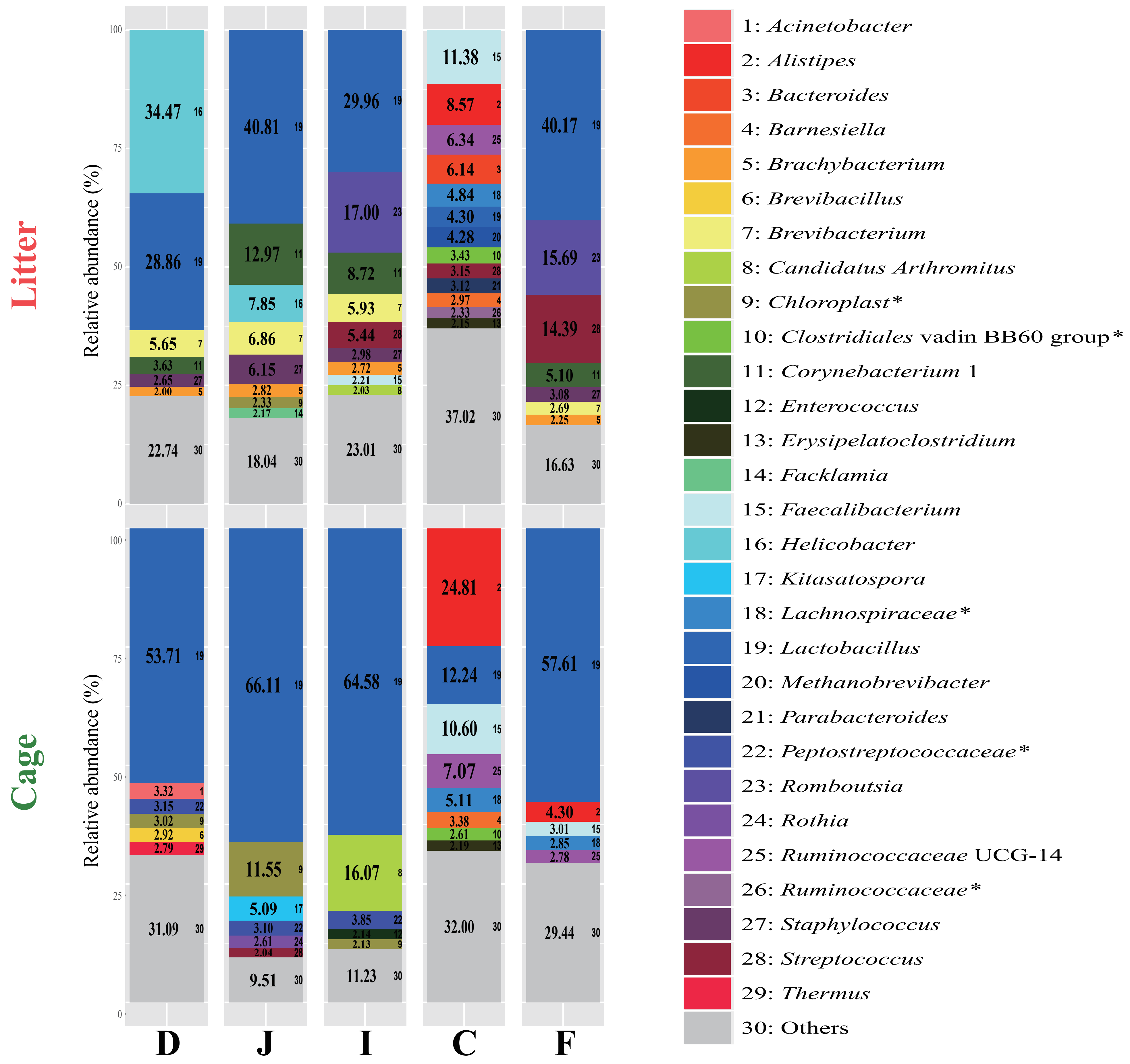

Supplementary Fig. 2 Average relative abundances of predominant genera in the litter and cage groups in different gut sites at 57 days of age. Only genera with relative abundances over 2% are shown with taxonomic annotations. D, J, I, C, and F denote duodenum, jejunum, ileum, cecum, and feces, respectively.

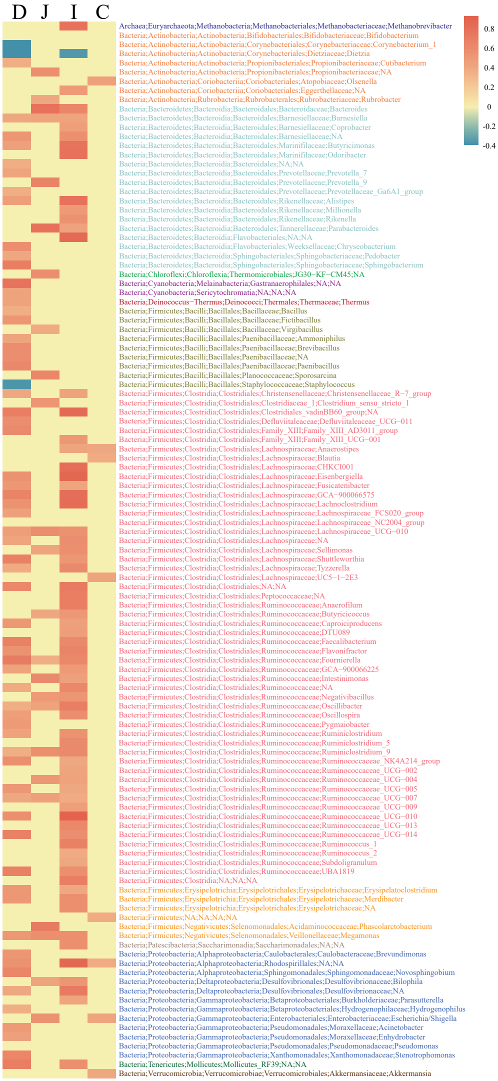

Supplementary Fig. 3 Associated with Fig. 4. Heatmap of correlations between *Campylobacter* and other microbes in different intestinal segments. Only significant correlations ($P < 0.05$) over 0.3 or below -0.25 are shown.

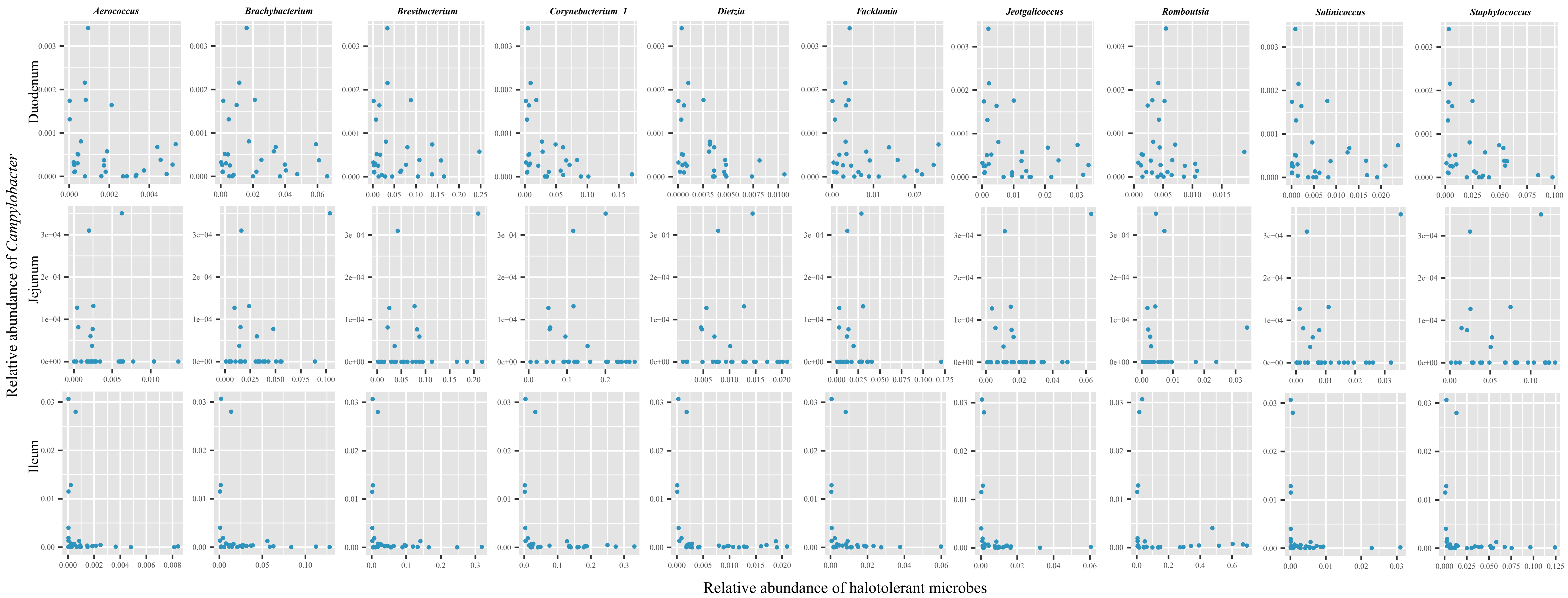

Supplementary Fig. 4 Nonlinear relationships between *Campylobacter* and genera that are halotolerant and aerobic or facultative anaerobic in the small intestine.

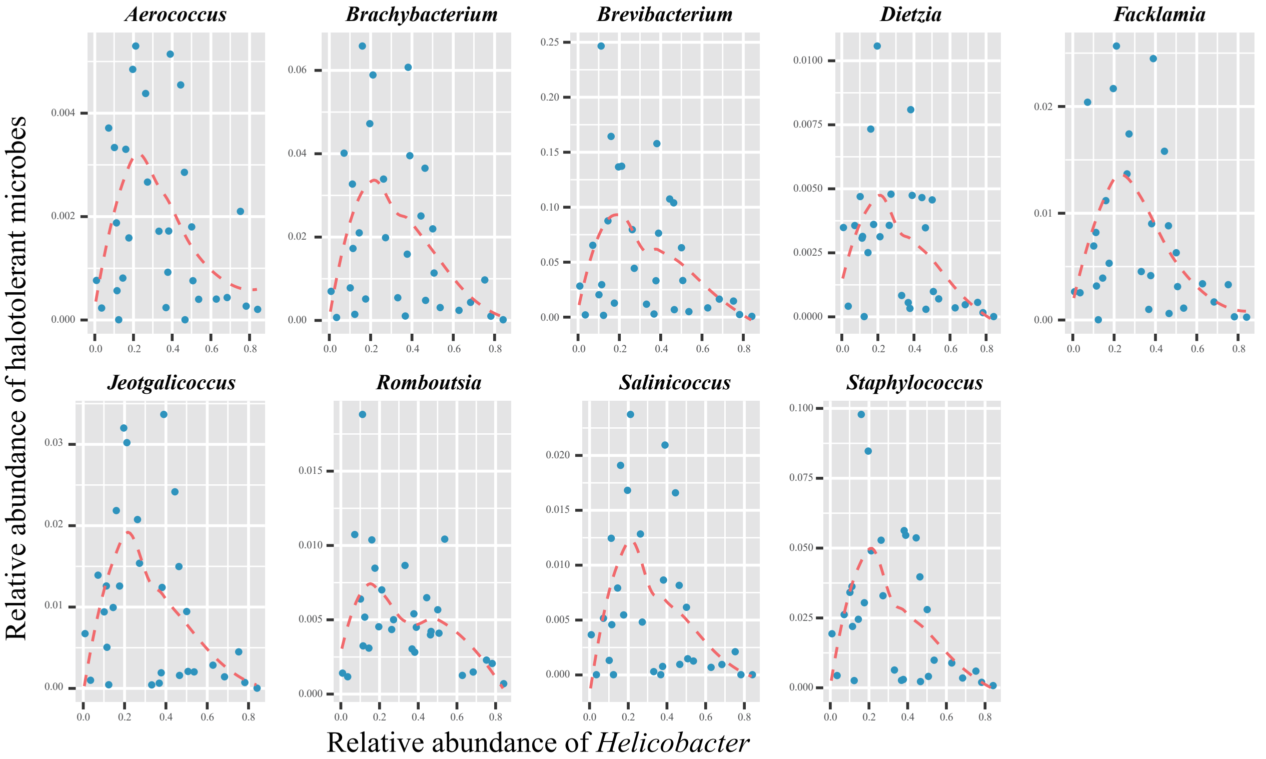

Supplementary Fig. 5 Nonlinear relationships between *Helicobacter* and genera that are halotolerant and aerobic or facultative anaerobic in duodenum.

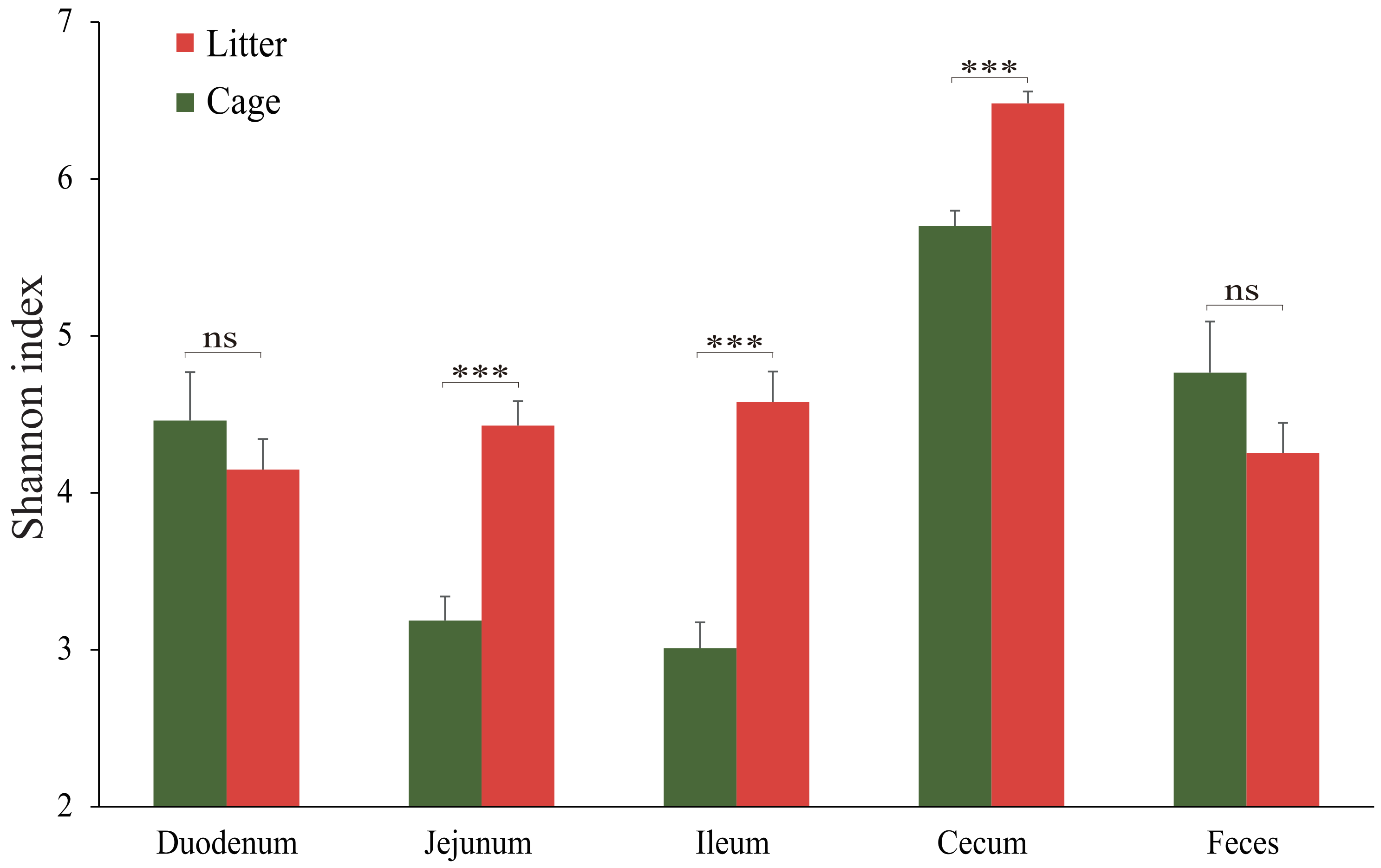

Supplementary Fig. 6 The Shannon index between the litter and cage groups in different gut sites at 57 days of age.
